# Idiopathic Pulmonary Fibrosis (IPF): An Overview

**DOI:** 10.3390/jcm7080201

**Published:** 2018-08-06

**Authors:** Shaney L. Barratt, Andrew Creamer, Conal Hayton, Nazia Chaudhuri

**Affiliations:** 1 Bristol Interstitial Lung Disease Service, North Bristol NHS Trust, Bristol BS10 5NB, UK; andrew.creamer@nbt.nhs.uk; 2 Academic Respiratory Unit, University of Bristol, Bristol BS16 1QY, UK; 3 North West Interstitial Lung Disease Unit, Manchester University NHS Foundation Trust, Wythenshawe, Manchester M23 9LT, UK; conal.hayton@nhs.net (C.H.); nazia.chaudhuri@nhs.net (N.C.)

**Keywords:** idiopathic pulmonary fibrosis, interstitial lung disease, nintedanib, pirfenidone

## Abstract

Idiopathic pulmonary fibrosis (IPF) is an interstitial lung disease characterised by chronic, progressive scarring of the lungs and the pathological hallmark of usual interstitial pneumonia. Current paradigms suggest alveolar epithelial cell damage is a key initiating factor. Globally, incidence of the disease is rising, with associated high morbidity, mortality, and economic healthcare burden. Diagnosis relies on a multidisciplinary team approach with exclusion of other causes of interstitial lung disease. Over recent years, two novel antifibrotic therapies, pirfenidone and nintedanib, have been developed, providing treatment options for many patients with IPF, with several other agents in early clinical trials. Current efforts are directed at identifying key biomarkers that may direct more customized patient-centred healthcare to improve outcomes for these patients in the future.

## 1. Introduction

The interstitial lung diseases (ILDs) are a heterogeneous group of parenchymal lung diseases characterised by varying degrees of inflammation and fibrosis. Some of these may occur secondary to a known precipitant such as drugs, autoimmune connective tissue disease, hypersensitivity to inhaled organic antigens, or sarcoidosis, whilst others, the idiopathic interstitial pneumonias (IIPs), have no identifiable cause [1]. Idiopathic pulmonary fibrosis (IPF) is one of the most aggressive forms of IIP, characterised by chronic, progressive fibrosis associated with inexorable decline in lung function, progressive respiratory failure, and high mortality. Accurate diagnosis is essential to help with prognostication and optimise treatment selection. This review will discuss the diagnostic criteria of IPF, our current understanding of disease pathogenesis (including the role of genetic and environmental factors in disease initiation and progression), current treatment modalities, and future therapeutic perspectives.

## 2. Epidemiology

IPF is the most common form of ILD. Reported incidence rates for IPF vary considerably depending on the method of data collection and diagnostic case definition. A systematic review of the global incidence of IPF estimated a rate of 2.8–9.3 per 100,000 per year in North America and Europe with significantly lower rates in Asia and South America. Regional variation within countries has also been observed, possibly reflecting exposure to environmental or occupational risk factors [2,3,4,5,6]. Evidence suggests that the incidence of IPF is rising [7]. A recent analysis of a UK-based primary care data-base calculated a rise in incidence of 78% between 2000 and 2012, as well as a doubling of prevalence, estimated at 38.8 per 100,000 [5], with the consequential growing economic burden on global health care [8].

Mortality in IPF is high, with a reported median survival of 2–3 years from diagnosis, based on historical data [9]. More recent evidence shows no improvement in survival [3,5,10]. Mortality rates also appear to be rising, although this may partly reflect increased recognition and diagnosis [2,11,12]. Over the last five years, antifibrotic therapies have become increasingly available, and at present the global impact of this on survival in IPF is unclear. Early evidence from an open label extension of the pirfenidone clinical trials reported an on-treatment median survival of 77.2 months [13]. It is well recognised that IPF is a heterogeneous disease with a variable disease course [14]. Predicting disease outcomes is difficult, particularly as baseline lung function alone is a poor predictor of mortality [15]. Composite scoring systems, such as the Gender-Age-Physiology (GAP) index [16], which include demographic and physiological parameters, may offer better prognostic accuracy. 

## 3. Diagnosis

International consensus guidelines recommend that the diagnosis of IPF be made at a multi-disciplinary level, requiring the exclusion of known causes of ILD and the presence of a usual interstitial pneumonia (UIP) pattern on high resolution computer tomography (HRCT) or surgical lung biopsy [9]. A radiological diagnosis of ‘definite UIP’ can be made based on HRCT alone in the presence of honeycomb cysts and subpleural, basally predominant reticulation, with or without traction bronchiectasis/bronchiolectasis, negating the need for surgical biopsy. Until recently, a radiological diagnosis of ‘possible UIP’ was used to describe the HRCT pattern where honeycomb cysts were notably absent but all other remaining features were present. In this circumstance guidelines suggested confirmation be sought through histological examination of surgical biopsy specimens [9]. In clinical practice, surgical biopsy rates are low due to concerns regarding associated morbidity and mortality, particularly in an elderly co-morbid patient group [17]. Evidence suggests that ‘possible UIP’ on HRCT, in the correct clinical context, has a high positive predictive value for UIP at biopsy [18,19]. It is therefore common for a “working diagnosis” of IPF to be made in the context of a typical presentation and the presence of ‘possible UIP’ on HRCT imaging [20,21]. In recognition of this requirement to adopt a practical approach to IPF diagnosis, the Fleischner Society published a position paper recommending the replacement of ‘possible UIP’ terminology with ‘probable UIP’, to describe the presence of reticulation and traction bronchiectasis/bronchiolectasis in a basal and subpleural distribution, in the absence of honeycombing [22]. They argue that this should be sufficient to support a diagnosis of IPF, reducing the need for surgical biopsy. It is anticipated that this approach will be incorporated into forthcoming IPF global guidelines. 

Despite evolution of diagnostic criteria, the process remains challenging and can be lengthy and stressful for patients [23]. There is a drive to improve diagnosis through technological innovation including cryobiopsy as an alternative to surgical lung biopsy [24], and computer-based CT image analysis to improve consistency of radiological interpretation which is known to be variable [25,26].

## 4. Pathogenesis

**Figure 1:**
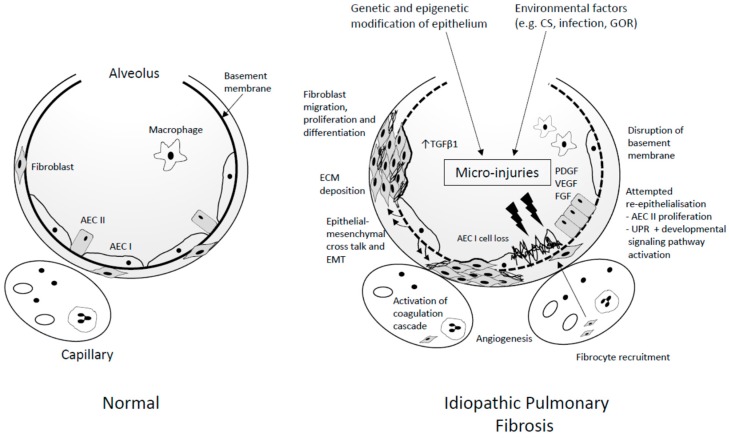
Schematic view of idiopathic pulmonary fibrosis (IPF) pathogenesis. Genetic and epigenetic phenomenon contribute to the development of an inherently dysfunctional epithelium. This epithelium is susceptible to recurrent micro-injury from environmental exposures (such as cigarette smoke (CS), inhaled dusts, infection, and gastro-oesophageal reflux (GOR)). The inability of the dysfunctional epithelium to regenerate following repetitive injury is a significant juncture in the propagation of IPF. Damage to the epithelium, disrupts the basement membrane and thus the alveolar capillary barrier. Capillary leakage of proteins (including fibrin and fibronectin) into the interstitial and alveolar spaces occurs, with activation of the coagulation cascade and abnormal vascular remodelling as part of the ongoing attempted repair process. Activated epithelial and endothelial cells create a milieu whereby aberrant epithelial–mesenchymal crosstalk, alongside fibrocyte/fibroblast recruitment, migration, proliferation, and differentiation, flourishes. Transforming growth factor β1 (TGFβ1), platelet-derived growth factor (PDGF), vascular endothelial growth factor (VEGF), and fibroblast growth factor (FGF) are some of the pro-fibrotic mediators implicated in these processes. Collections of active fibroblasts and myofibroblasts form fibrotic foci (FF), considered to be at the leading edge of extracellular matrix (ECM) deposition, with progressive lung remodelling and architectural distortion. AEC I/II: alveolar epithelial type I/II cell; UPR: unfolded protein response; EMT: epithelial–mesenchymal transition; UPR: unfolded protein response.

UIP is the characteristic histopathological hallmark of IPF. Features include temporal and spatially heterogeneous fibrosis, clusters of fibroblasts and myofibroblasts (fibroblastic foci), and excessive deposition of disorganised collagen and extracellular matrix (ECM), with resulting distortion of the normal lung architecture, with or without honeycomb cyst formation [27]. Whilst precise factors that initiate these processes are unknown, current paradigms suggest that IPF is the consequence of an aberrant repair process in response to complex interactions between both host and environmental factors (Figure 1). A comprehensive review of the pathogenesis of IPF is beyond the scope of this article and has been discussed recently elsewhere [28,29]. Key pathogenic concepts are outlined below.

### 4.1. Initiation

#### 4.1.1. Genetic Basis for IPF

Studies of familial interstitial pneumonia [30,31] and large-scale genome-wide association studies (GWAS) [32,33] have provided an abundance of evidence to support a genetic predisposition to pulmonary fibrosis.

#### 4.1.2. Surfactant Protein-Related Genes

The surfactant proteins are alveolar epithelial type II cell (AEC type II)-specific proteins considered to be essential for normal lung function and homeostasis. Variants of surfactant proteins C (SP-C) and A2 (SP-A2) have been implicated in the development of familial pulmonary fibrosis (FPF) [31,34,35,36] but only in rare cases of sporadic IPF [37,38].

Studies suggest that these mutations prevent correct folding of proteins within the endoplasmic reticulum (ER), leading to ER stress and initiation of the unfolded protein response (UPR) [39,40]. The UPR represents a series of protective biochemical pathways that aim to match the protein capacity of the ER. Failure to do so triggers cellular apoptosis [40,41], and in the case of the aforementioned surfactant protein mutations, AEC apoptosis ensues [42,43,44,45].

Interestingly, markers of UPR activation are also elevated in the alveolar epithelial type II cells of patients with IPF [44,46], although the exact mechanistic drivers remain largely undefined [39]. Furthermore, whether UPR activation is sufficient to lead to lung fibrosis is equally unclear [39]. In preclinical models of pulmonary fibrosis, activation of UPR for 6 months was insufficient to independently promote fibrosis in mice but altered the epithelial phenotype such that it was predisposed to stimulate downstream profibrotic pathways following exposure to a second insult [47].

#### 4.1.3. *MUC5B* Promoter Polymorphism 

The *MUC5B* promoter single-nucleotide polymorphism (SNP) has been identified as a strong risk factor for the development of both familial and sporadic idiopathic interstitial pneumonias. Odds ratios for disease in subjects heterozygous (GT) and homozygous (TT) for the minor allele of this *MUC5B* polymorphism (rs35705950) have been quoted as 6.8 (95% confidence interval (CI), 3.9–12.0) and 20.8 (95% CI, 3.8–113.7) for familial cases, and 9.0 (95% CI, 6.2–13.1) and 21.8 (95% CI, 5.1–93.5) for IPF [48], with validation in several independent cohorts [49,50], although the minor allele is present in 19% of non-Hispanic white populations, many without disease [51]. 

The role of this gene in the pathogenesis of IPF has not been fully elucidated, although mechanistic studies suggest that overexpression of MUC5B, driven by the promoter polymorphism, increases chronic mucus hypersecretion, impairs mucociliary clearance in the broncho-alveolar region, and potentiates chronic inflammation and injury [51]. Interestingly, the presence of the MUC5B promoter polymorphism not only appears to be predictive of pulmonary fibrosis but also prognostic, with an observed twofold improved survival in IPF cohorts expressing the polymorphism compared to non-carriers [52]. This suggests that increased mucin may provide enhanced host microbial defence [51].

#### 4.1.4. Other Gene Variants

GWAS have also identified three single nucleotide polymorphisms (SNPs) in the toll-interacting protein (*TOLLIP*) gene [32] in association with IPF development. Whilst the minor allele of rs5743890 protects against the development of IPF, it is also associated with increased mortality in IPF patients with established disease [52].

#### 4.1.5. Telomeres

Telomeres are specialised loop structures of repeating 5′-TTAGGG-3′ DNA units and associated binding proteins (the shelterin complex) that cap the tips of chromosomes, protecting chromosomal DNA from degradation. Progressive shortening of the telomere occurs during each cycle of DNA replication until a critical telomere length is reached, triggering cell senescence or chromosomal degradation and cell death [53]. Telomerase is an enzyme consisting of protein (hTERT) and RNA (hTR) subunits that add 5′-TTAGGG-3′ repeats to the tips of chromosomes during DNA replication, thus preventing progressive telomere shortening. It is active in germline and stem cells but is extremely low or absent in normal somatic cells [54]. 

Accelerated telomere shortening has been associated with premature ageing and abnormal tissue repair [55]. In preclinical models, telomere dysfunction, studied in telomerase-deficient mice [56] or through the disruption of the shelterin complex formation in ATII cells of mice [57], both induce the formation of pulmonary fibrosis.

Six telomere-related genes have been linked to FPF (Table 1); *TERT* [30], *TERC* [58], *DKC1* [59], *TINF2* [60], *RTEL1* [61], and *PARN* [62], although *TERT* mutations are most frequent, occurring in approximately 15% of those with affected kindreds [63].

There appears to be poor genotype-ILD phenotype correlation across patients with telomere-related gene mutations and indeed within families that have the same genetic mutation [64,65]. Cases of idiopathic interstitial pneumonias of unknown cause (nonspecific interstitial pneumonia, desquamative interstitial pneumonia, pleuroparenchymal fibroelastosis, and IPF) as well as ILD attributed to known causes (chronic hypersensitivity pneumonitis and connective tissue disease-associated ILD) have been identified in those with telomere-related gene mutations [64,66]. Despite the varied ILD presentations associated with these gene mutations, a consistent phenotype of progressive disease manifests, irrespective of the radiological or histological pattern of ILD [64], with increased risk of severe haematological complications after lung transplantation [66]. Furthermore, shorter circulating leucocyte telomere length in IPF is also independently associated with reduced transplant free survival [67].

The concept of removing senescent cells as a form of therapy for those with telomere-dysfunction or age-related diseases is a rapidly evolving area of research. Genetic deletion of specific senescent cells (p16^INK4a^ positive) and similarly the use of pharmacological drivers of senescent cell apoptosis, improved lung function and fitness of animals in pre-clinical models of pulmonary fibrosis [68]. Navitoclax, an inhibitor of the Bcl-2 family, has been shown to reverse pulmonary fibrosis induced by thoracic irradiation of mice [69], whilst gestelmir (a dyskerin internal peptide) induces telomerase activity, restoring telomere length and decreasing aging in cells of those with congenital dyskeratosis [70]. These may be viable options to explore novel approaches to the pharmacological management of IPF/ILD in the future, but consideration as to the consequence of anti-senescence in a condition with increased cancer risk should be given.

### 4.2. Environmental Factors

As previously discussed, genetic phenomena are thought to contribute to the development of an inherently dysfunctional epithelium. Current paradigms suggest that this epithelium is then susceptible to recurrent micro-injury from several potential environmental sources [39], a hypothesis that is supported by the temporal and spatially heterogeneous features of the pathological hallmark of this disease.

Epidemiological studies have highlighted occupational and environmental exposures to wood and metal dust, pollution, gastric aspiration, smoking, and infection as factors that may confer an increased risk of developing IPF [71].

CS is the most prevalent environmental exposure linked with the development of IPF [72], with evidence supporting a self-sustaining pathological response that continues despite smoking cessation [73]. One possible mechanistic link could be through the effect of CS on the epigenome. Studies have shown that CS increases methylation of specific gene promoters involved in the pathogenesis of IPF, such as *WNT7A* [74] and alters DNA methylation [75], histone modification [76], and micro-RNA expression [77,78]. 

Viral pathogens have also been implicated in the initiation of IPF. The human herpes virus (HHV) family have been the most widely studied, albeit in small, retrospective studies [79,80,81,82,83,84]. In one cohort of IPF patients, past infection with at least one HHV occurred in 97% patients compared to 36% healthy controls [79], whilst several other studies have consistently reported findings of increased Epstein Barr virus in bronchoalveolar lavage and lung specimens compared to controls [81,82,83]. HHV induction of ER stress and AEC apoptosis via the UFR has been proposed as a potential mechanism [44,85].

A role for bacteria in the initiation and progression of IPF has developed as a more novel concept in recent years, challenging current paradigms of acute exacerbations of IPF (AE-IPF), that by definition requires the exclusion of an infective trigger [86]. In a study by Molyneaux et al. [87], AE-IPF was associated with increased BAL bacterial burden compared to stable IPF, with a notable change in the microbiota following an AE-IPF.

Shulgina et al. evaluated the benefit of 12 months of prophylactic co-trimoxazole in patients with IPF in a large multicentre, randomised controlled trial [88]. Although there was no effect on change in forced vital capacity (primary endpoint) compared to placebo, there were reductions in infections and mortality in co-trimoxazole-treated individuals in a post hoc analysis. The EME-TIPAC trial (Efficacy and Mechanism Evaluation of Treating Idiopathic Pulmonary fibrosis with the Addition of Co-trimoxazole) will determine whether co-trimoxazole is efficacious in terms of reducing mortality and/or hospitalisation and should also provide some insight into whether its antibacterial properties prove an important mechanism of action in IPF [89].

There is growing consensus on the role of chronic gastric fluid microaspiration as the initial AEC insult driving aberrant tissue remodelling in IPF [90]. In pre-clinical studies, aspiration of gastric fluid was shown to activate fibrotic cascades in the pulmonary parenchyma [91]. Furthermore, elevated BAL pepsin, a biomarker for gastric aspiration, was found in AE-IPF compared to stable IPF patient populations, suggesting gastro-oesophageal microaspiration may be a trigger for AE-IPF [92]. The presence of *Campylobacter* in the AE-IPF microbiota strengthens this association [92].

A recent meta-analysis suggested pharmacological management of gastro-oesophageal reflux (GER) was associated with a significant reduction in IPF-related mortality, although these conclusions were drawn from observational studies in the absence of any available RCT data [93]. Indeed, a causal relationship between GER and IPF has yet to be formerly established; some groups suggest that increased reflux of gastric contents may simply represent the consequence of reduced lung compliance, distorted mediastinal anatomy and weakening of the lower oesophageal sphincter [94,95].

### 4.3. Propagation

The inability of the dysfunctional epithelium to regenerate following repetitive injury is a significant juncture in the propagation of IPF [28,96]. Damage to the epithelium disrupts the basement membrane and thus the alveolar capillary barrier [97]. Capillary leakage of proteins (including fibrin and fibronectin) into the interstitial and alveolar spaces occurs [98], with activation of the coagulation cascade [99] and abnormal vascular remodelling as part of the ongoing attempted repair process [100,101]. Activated epithelial and endothelial cells create a milieu whereby aberrant epithelial–mesenchymal crosstalk, alongside fibrocyte/fibroblast recruitment, migration, proliferation, and differentiation, flourishes.

Expansion of the resident fibroblast population is thought to be the predominant source of collagen producing cells in the lung during the development of fibrosis [102], but bone marrow derived fibrocytes may also comprise a small population of cells that expand and migrate to sites of injury [103].

Epithelial–mesenchymal transition (EMT) is the process by which epithelial cells acquire phenotypic characteristics of mesenchymal cells [104], a process requiring considerable alteration in transcriptional machinery and cellular reprogramming. Whilst EMT is a physiological phenomenon in embryological tissue, it is a rare event in normal wound healing and occurs in response to sustained inflammation and injury [105,106]. Transforming growth factor β1 (TGF β) is thought to be a key cytokine involved in the initiation of this process [106]. Whilst there is evidence to suggest that epithelial cells acquire some mesenchymal features in IPF, it remains unclear as to whether formal EMT with transition of AEC to fibroblasts occurs, partly as there are no definitive specific fibroblast cell markers [107]. Nevertheless, collections of active fibroblasts and myofibroblasts form fibroblastic foci (FF), considered to be at the leading edge of exaggerated ECM deposition [108], with progressive lung remodelling, architectural distortion, and fibrosis [28,109].

## 5. Treatment

The treatment approach to IPF has evolved considerably over the last two decades. A typical regimen in the year 2000 involved immunosuppression with prednisolone and azathioprine (2000). The Idiopathic Pulmonary Fibrosis International Group Exploring N-Acetylcysteine I Annual (IFIGENIA) study [110], published in 2005 suggested that the addition of N-acetylcysteine (NAC), an anti-oxidant compound, to this combination would help to preserve lung function. However, the Prednisolone, Azathioprine, and N-Acetylcysteine: A Study That Evaluates Response (PANTHER) study [111] found that patients taking this triple combination therapy were at increased risk of death and hospitalisation compared to patients receiving placebo alone. Furthermore, as compared with placebo, NAC in isolation offered no significant benefit with respect to the preservation of forced vital capacity (FVC) in patients with IPF compared to placebo [112]. This led to a shift away from immunosuppression and NAC, leaving a treatment gap with no apparent effective option available. However, over the last 5 years, two novel antifibrotic therapies, pirfenidone and nintedanib, have been developed, providing treatment options for many patients with IPF [113].

Pirfenidone is a novel compound with anti-inflammatory and anti-fibrotic properties [114,115]. Early phase II and III studies in Japan identified it as a potential therapeutic option in IPF [116,117]. This was followed internationally by the Clinical Studies Assessing Pirfenidone in idiopathic pulmonary fibrosis: Research of Efficacy and Safety Outcomes (CAPACITY) trials (PIPF-004 and PIPF-006) [118], concurrent randomised control trials (RCT) in IPF comparing pirfenidone at doses of 2403 mg/day and 1197 mg/day against placebo over 72 weeks. In the PIPF-004 trial, pirfenidone at the 2403 mg/day dose achieved a significant reduction in FVC decline. Although this was not matched in the PIPF-006 trial, a pre-specified pooled analysis identified a significant treatment effect with an 8% decline in FVC in the pirfenidone group compared to 11% receiving placebo. Significantly fewer patients suffered a decline in FVC of 10% or greater and progression-free survival was improved by 26%. A further phase III trial, the Assessment of Pirfenidone to Confirm Efficacy and Safety in Idiopathic Pulmonary Fibrosis (ASCEND) study [119], was requested by the United States Food and Drug Administration (US FDA) due to discrepancies between the two CAPACITY trials in meeting their primary endpoints. In this study 555 patients with IPF were randomised to receive pirfenidone (2403 mg/day) or placebo for 52 weeks. Treatment with pirfenidone led to a significant reduction in the proportion of patients suffering disease progression (absolute decline in FVC of ≥10% or death), which was the primary endpoint. Pooled analysis of the CAPACITY and ASCEND studies found that treatment with pirfenidone at 2403 mg/day reduced the proportion of patients experiencing a FVC of ≥10% or death by 43.8% [120]. In addition, there was a reduction in the relative risk of all-cause and IPF-related mortality at 52 weeks with pirfenidone treatment [121]. Post-hoc analysis indicates that the efficacy of pirfenidone is independent of baseline disease severity or demographics [120,122]. 

Nintedanib, a tyrosine kinase inhibitor initially developed as an anti-tumour agent [123], was noted to have activity against fibroblasts through inhibition of vascular endothelial growth factor (VEGF) and other profibrotic mediators such as platelet-derived growth factor (PDGF), fibroblast growth factor (FGF), and transforming growth factor (TGF)-β [124,125]. An initial phase II study, the To Improve Pulmonary Fibrosis with BIBF 1120 (TOMORROW) study [126], suggested that nintedanib at a dose of 150 mg twice daily was effective at reducing FVC decline although failed to reach statistical significance. Subsequently, the INPULSIS trials (INPULSIS-1 and 2) [127], parallel phase III, multicentre RCTs, demonstrated a significant reduction in the rate of FVC decline over a 52-week period, in IPF patients receiving nintedanib compared to placebo. In the pooled analysis, the mean difference in FVC decline was 109.9 mL/year and significantly fewer patients suffered a 5 or 10% decline in FVC with nintedanib. No mortality benefit was noted with nintedanib treatment in the INPULSIS trials or when the data was pooled with the TOMORROW study [128]. Evidence from TOMORROW and INPULSIS-2 suggests that nintedanib may reduce the frequency of acute exacerbations of IPF however this was not observed in INPULSIS-1 [126,127] and there was no evidence from pooled data that mortality post-exacerbation improved with nintedanib [129]. In similarity to pirfenidone, post-hoc analysis has suggested that nintedanib is equally efficacious in patients with mild or severe disease [130,131], irrespective of baseline characteristics [130] and appears to be equally effective in patients with a ‘possible UIP’ pattern compared to those with ‘definite UIP’ radiologically [132].

Meta-analysis indicates similar efficacy between both antifibrotics [133,134]. Treatment decisions are generally driven by tolerance to side effect profiles [135]. Adverse events were common in all the major anti-fibrotic studies with discontinuation rates of 11.9% for pirfenidone in the CAPACITY and ASCEND studies and 19.3% for nintedanib in INPULSIS [120,127]. Gastrointestinal side effects are common with both antifibrotics, with high rates of diarrhoea (62.4%) reported with nintedanib in INPULSIS, while a photosensitivity rash was observed in 29.2% of patients receiving pirfenidone. Both drugs reported higher proportions of patients suffering a rise in liver function tests in comparison to placebo. Despite frequency of adverse events, results from real-world studies suggest that both drugs are well tolerated in practice [136,137,138,139,140,141], particularly with support to manage side effects [142]. Recent studies have assessed combination therapy with both antifibrotics and have reported adequate tolerability with promising secondary efficacy data [143,144]. 

## 6. Future Perspectives

### 6.1. Personalised Medicine

The IPF disease course and response to anti-fibrotic therapy demonstrates marked heterogeneity, making individual prognostication difficult. Progression towards more customized patient-centred healthcare is essential to achieve better outcomes in the future [145,146].

Many biological markers or biomarkers have been explored with respect to predicting prognosis or treatment response in IPF, with several blood biomarkers showing promise with regards to the emerging concept of precision medicine [147,148]. Whilst it is beyond the scope of this review to discuss all the potential biomarkers cited in the literature, several blood biomarkers showing promise with regards to the emerging concept of precision medicine are highlighted. 

The matrix metalloproteinases (MMPs) are proteases involved in the remodelling of extracellular matrix components [149]. MMP-7 appears to be one of the most interesting candidates with regards to a single biomarker. Circulating levels of MMP-7 are consistently elevated in the blood of patients with IPF compared to controls [150,151,152,153], with significant correlation between higher MMP-7 levels and IPF disease severity as assessed by FVC and D_LCO_ (% predicted) [150]. Furthermore, the rate of increase of breakdown products of MMP activity also predicted worse IPF survival [154,155].

The alveolar epithelial markers surfactant protein-D (SP-D) and Krebs von den Lungen-6 (KL-6), also known as mucin 1 (MUC-1), have been widely used in clinical practice in Japan as part of the diagnostic work-up for ILD for more than 10-years, although evidence from clinical trials validating their clinical efficacy in IPF remains limited [156]. Significantly elevated circulating levels of both SP-D and KL-6 have been consistently found in IPF patients compared to controls [156,157,158,159] but with regards to prognostication, neither SP-D nor KL-6 improved the prediction of survival beyond a clinical model of age, forced vital capacity (FVC), lung CO-diffusing capacity (D_LCO_), and change in FVC [160]. Furthermore, neither SP-D [117,147] nor KL-6 [147,161] significantly reduced following initiation of anti-fibrotic therapy.

Given the complexity of IPF disease pathogenesis it is unlikely that a single biomarker will prove to be sufficiently specific to IPF and also be able to predict disease evolution; a panel of biomarkers is likely to be more successful. Gene expression profiling of several IPF cohorts has identified a collection of 52 specific peripheral blood mononuclear cell genes that when interpreted together predicts poor outcomes in IPF [152,162]. Alterations in this gene expression pattern were also noted in a subset of patients with stabilisation of FVC upon treatment with anti-fibrotic medications [152]. Although further work is required to independently validate this dataset and establish robust normal reference ranges for the gene signature before it can be trialled clinically, it provides an exciting step towards the delivery of more personalised healthcare.

Internationally it has been recognised that to drive the concept of ‘patient-centred healthcare delivery’ in the future, an investigational approach of integrated systems biology is required, bringing together cellular, molecular, clinical, and environmental research to provide a more comprehensive understanding of disease development, progression and response to therapy [163]. 

### 6.2. Novel Therapeutic Targets

Fibrogenesis represents the final common pathway of disequilibrium in several processes that represents the complex interplay of cellular mechanisms, growth factors, cytokines and signalling pathways.

**Figure 2:**
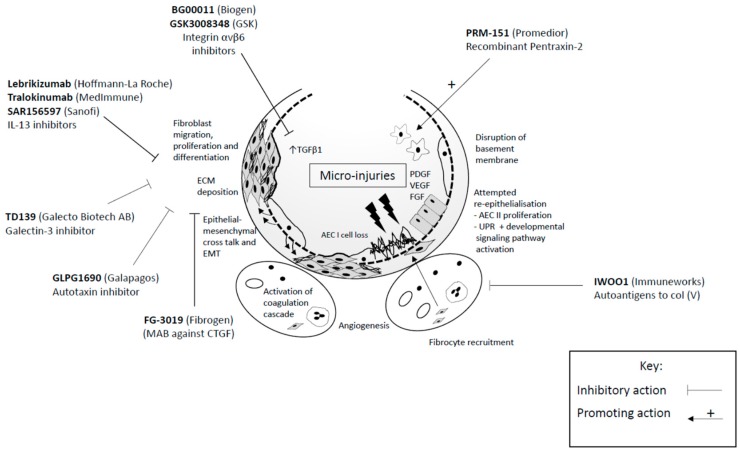
Novel therapeutic agents in idiopathic pulmonary fibrosis (IPF). Growing understanding of the pathogenesis of IPF has enabled the identification of a variety of putative molecular targets and has led to the development of multiple novel agents, many of which are in early clinical trials. Several of these agents and their mode of action are highlighted above. Please refer to text for full description of action. Abbreviations: MAB: monoclonal antibody; CTGF: connective tissue growth factor; Col (V): type V collagen; IL-13: interleukin-13. The lead pharmaceutical company for each agent is highlighted in brackets.

Growing understanding of the pathogenesis of IPF has enabled the identification of a variety of putative molecular targets and has led to the development of multiple novel agents, many of which are in early clinical trials. Whilst a comprehensive review of all emerging therapies in IPF is beyond the scope of this article, we focus on recent and exciting advancements (Figure 2).

#### 6.2.1. Growth Factors and Associated Signalling Pathways

Connective tissue growth factor (CTGF) is normally expressed at low levels in healthy adults, but when expressed in excess it is thought to co-ordinate the upregulation of several pro-fibrotic growth factors (including TGF-β1), promote ECM production, and inhibit ECM degradation [36]. FG-3019 (Fibrogen, Birmingham, AL, USA, NCT 01890265) is a humanised monoclonal antibody (MAB) directed against CTGF that is currently undergoing phase II randomised controlled trial assessment, having demonstrated good safety profiles and promising outcomes in recent open label study [164]. 

In addition to targeting the circulating growth factors themselves, the corresponding cell surface receptors are also potential targets. Integrin αvβ6, a key mediator of TGF-β activation, is known to be involved in the regulation of fibrogenesis and epithelial injury [165]. In a murine model of bleomycin-induced pulmonary fibrosis, partial inhibition of αvβ6 was found to effectively inhibit TGF-β activation, epithelial injury, and tissue fibrosis [166]. BG00011, formerly known as STX-100 (Biogen, Weston, MA, USA, NCT01371305), is a humanized MAB against integrin αvβ6 being investigated in escalating subcutaneous doses to assess its safety and tolerability, whilst GSK3008348 (GlaxoSmithKline, London, UK, NCT02612051) is being developed as the first inhaled inhibitor of αvβ6 integrin, having been well tolerated in phase I trials [167].

Autotaxin is an enzyme that plays a central role in the production of bioactive lysophosphatidic acid (LPA). Levels of LPA and autotaxin have been found to be elevated in bronchoalveolar lavage fluid and exhaled breath condensate in patients with IPF, with several reports suggesting a role of the autotaxin-LPA pathway in fibrogenesis [168,169,170]. GLPG1690 (Galapagos, Mechelen, Belgum, NCT02738801) is a novel, selective autotaxin inhibitor that has been shown to stabilize FVC in IPF patients following 12 weeks of treatment, compared to placebo, in a recently published phase IIa clinical trial (FLORA), in the absence of any safety or tolerability concerns [171]. A global phase III trial is due to start in 2018 (Isabela trials). 

#### 6.2.2. Cytokines

Interleukin-13 (IL-13) is a T-helper type 2 (Th2) cell cytokine that promotes lung fibrogenesis in a number of experimental settings [172,173,174]. Targeting of IL-13 in pre-clinical studies therapeutically blocks aberrant lung remodelling and promote repair [174]. Three MAB inhibitors of IL-13 are currently in phase II clinical trial development; lebrikizumab (Hoffmann-La Roche, Basel, Switzerland, NCT01872689), tralokinumab (MedImmune LLC, Gaithersburg, MD, USA, NCT01629667), and SAR156597 (Sanofi, Paris, France, NCT02345070), the latter a bispecific monoclonal antibody against both IL-4 and IL-13. Data from these trials is awaited.

#### 6.2.3. Alveolar Monocytes

Monocytes and macrophages have a key role in regulating tissue repair and fibrosis, and several molecular pathways regulating their activity are being investigated as potential treatments in IPF. Pentraxin-2 (PTX-2), also known as serum amyloid P, is a circulating protein which binds to Fc-gamma receptors on monocytes and promotes epithelial healing (Cox, Pilling, and Gomer 2014). Low circulating levels of PTX-2 have been found in IPF [175]. PRM-151 (Promedior, Lexington, MA, USA, NCT02550873) is a recombinant form of PTX-2, found to be safe and well tolerated in phase I trials [176,177]. It is currently being studied in a phase II placebo-controlled RCT (NCT02550873). 

Galectin-3 is another circulating protein which plays a key role in fibrosis development through the activation of macrophages and myofibroblasts [178,179]. TD139 (Galecto Biotech AB, Copenhagen, Denmark, NCT02257177) has been developed as a specific inhibitor of the galactoside binding pocket of galectin-3. It is administered via inhalation and is currently being investigated in a phase I trial.

#### 6.2.4. Developing Tolerance to Autoantigens

There is growing evidence that an adaptive immune response to exposed autoantigens, notably collagen proteins, may play a role in the progression of IPF [180]. Specifically, up to 60% of IPF patients have anti-type V collagen (Col (V))-reactive T cells [181] and nearly half develop specific systemic antibody responses [182]. Inducing tolerance to these autoantigens may therefore suppress the aberrant immune response. IWOO1 (Immuneworks, Indianapolis, IN, USA, NCT01199887) is an oral treatment designed to induce immune tolerance to the Col (V) protein in antibody-positive IPF patients. A phase I study of this agent reported no adverse effects, and results demonstrated a trend towards stabilisation of FVC in the highest-dose cohort [183]. 

## 7. Concluding Remarks

Whilst progress has been made over recent years to improve our understanding of this condition and provide therapeutic options, there is still much work to be done to identify key targets that may allow preventative interventions and to develop strategies and/or biomarkers to aid early diagnosis or even to suspend fibrogenesis in this devastating condition.

## Figures and Tables

**Table 1:** Telomere-related genes currently linked to familial pulmonary fibrosis. Six telomere-related genes have been linked to familial pulmonary fibrosis (FPF): *TERT* [30], *TERC* [58], *DKC1* [59], *TINF2* [60], *RTEL1* [61], and *PARN* [62], although *TERT* mutations are most frequent, occurring in approximately 15% those with affected kindreds [63].

Gene	Name	Function
*TERT*	Telomerase reverse transcriptase	Encodes the protein subunit of telomerase (hTERT)
*TERC*	Telomerase RNA component	Encodes the RNA subunit of telomerase (hTR)
*DKC-1*	Dyskerin pseudouridine synthase 1	Active role in telomerase stabilisation
*TINF2*	TRF1 (Telomerase repeat factor 1) interacting nuclear factor 2	Provides instructions of making part of the shelterin protein complex
*RTEL1*	Regulator of telomere elongation helicase 1	Encodes a DNA helicase that helps stabilize telomeres during RNA replication
*PARN*	Poly(A) specific ribonuclease	Involved in TERC RNA maturation
